# Depressive and Anxiety Symptom Assessment in Adults with Polycystic Ovarian Syndrome

**DOI:** 10.1155/2021/6652133

**Published:** 2021-04-17

**Authors:** Wadha K. Almeshari, Alanoud K. Alsubaie, Reham I. Alanazi, Yara A. Almalki, Nazish Masud, Sami H. Mahmoud

**Affiliations:** ^1^College of Medicine, King Saud bin Abdulaziz University for Health Sciences, Riyadh, Saudi Arabia; ^2^King Abdullah International Medical and Research Center, Riyadh, Saudi Arabia; ^3^Research Unit, College of Medicine, King Saud bin Abdulaziz University for Health Sciences, Riyadh, Saudi Arabia; ^4^Department of Psychiatry, King Saud bin Abdulaziz University for Health Sciences, Riyadh, Saudi Arabia

## Abstract

**Background:**

Polycystic ovarian syndrome (PCOS) is an endocrinopathic disorder commonly affecting women in the reproductive age. These women have a possibility of developing depression and anxiety due to biochemical changes, concerns regarding physical appearance, and social pressure from infertility. Thus, the connection between PCOS, anxiety, and depression has a possible impact on patients' quality of life. This study is aimed at assessing depression and anxiety symptoms among PCOS patients and their association with different socioeconomic aspects.

**Methods:**

A cross-sectional study was conducted to assess depression and anxiety symptoms on 250 PCOS patients which were selected through consecutive sampling technique. Arabic versions of the HAM-A and HAM-D questionnaires were used alongside a demographic sheet to determine the socioeconomic and fertility status.

**Results:**

Prevalence of anxiety symptoms was reported among 100 (40%) of women and was found to be significantly higher in single women with a prevalence of 59 (48%) (*χ*2 = 5.8, *p* value <0.01). Also, lower-income status and unemployment were associated with a significantly higher prevalence of anxiety 18 (67%) (*χ*2 = 10.3, *p* value =0.03) and 71 (45%) (*χ*2 = 4.5, *p* value =0.03) women, respectively. Depressive symptoms were reported among 122 (49%) participants.

**Conclusion:**

Single marital status, low income, and unemployment were predictors of anxiety. Tension was noted to be the most common anxiety symptom among participants while depressed mood and psychological anxiety were the most reported depressive symptoms. It is important to note the link between anxiety, PCOS, and depression when deciding treatment plans for affected women.

## 1. Introduction

Polycystic ovarian syndrome (PCOS) is an endocrinopathic disorder commonly affecting women in the reproductive age group [1]. It is the most common endocrine disorder; its prevalence ranges from 2.2% to 26.7% worldwide [2]. In Saudi Arabia, the prevalence of PCOS has not been yet identified; however, 82% of women presenting with hirsutism are found to have PCOS [3]. Its diagnostic criteria include chronic oligo or anovulation, defined as irregular or none occurring menstrual cycles, presence of ovarian cysts on ultrasound examination, and high levels of androgen hormones [4]. Increasing androgen levels causes alopecia, acne, and hirsutism, which can affect physical, social, and emotional wellbeing of women [5].

Many studies have shown the association of depression and anxiety with PCOS. One which was conducted in China showed the prevalence of depression and anxiety among PCOS patients to be 27.5% and 13.3%, respectively [6]. Moreover, a study conducted on adolescents with PCOS in Turkey found the prevalence of depression to be significantly higher [7]. The psychological effect of PCOS is found to be due to its physical manifestations and symptoms, such as obesity, hirsutism, and acne, which lead to low self-esteem and dissatisfaction regarding the physical appearance [8]. The biochemical effect is due to androgen excess and abdominal obesity, leading to dyslipidemia in some cases and other metabolic impairments, but the direct mechanism is not determined yet [8, 9]. PCOS patients experience anovulation and menstrual irregularity leading to fertility difficulties after marriage, which is one of the major reasons for social and family pressures leading to depression and anxiety [10].

The current study is aimed at evaluating the prevalence of depressive and anxiety symptoms among women with PCOS within the child bearing age group. Furthermore, its goal was to understand the association between different socioeconomic aspects such as marital status, number of children, infertility, and income with the presence and severity of different depressive and anxiety symptoms, due to such factors being highly impactful on a person's psychological well-being. Moreover, the matters of marriage and childbearing are of a higher concern for affected women since they are considered to be emotional, instinctive, and social needs [11]. Assessment of the abovementioned aims would lead to more future awareness regarding the magnitude of PCOS psychological effects and the patients' concerns that needs to be addressed at the time of diagnosis, which in turn will modify treatment plans to include psychological screening, counseling, and psychiatric help as part of PCOS management [12].

## 2. Subjects and Methods

Ethical approval of this study was obtained from the IRB/0355/19 on 12th of March 2019, and written consent was obtained from the participants to assure their confidentiality.

### 2.1. Study Design and Settings

A cross-sectional study was conducted on women with PCOS to assess depression and anxiety symptoms. This study was conducted at gynecology clinics at Khashm Al-An primary health care center in King Abdulaziz Medical City in Riyadh, a governmental center for eligible and dependent people who had first-degree relatives working in Saudi Arabian National Guard or National Guard Health Affairs facilities.

### 2.2. Participants

To identify the potential participants, gynecology clinics were visited on weekly basis to check the list of appointments and determine all PCOS patients. While waiting for their appointments, women with PCOS were met and given the surveys by coinvestigators after informed written consent. The participants who were included in this study had to be in the 18 to 50 years old age group, with no chronic conditions other than PCOS. Pregnant women, those with diagnosed psychiatric disorders, acute illnesses, or who are being administered any psychiatric medications were excluded. Also, participants who experienced a recent unpleasant event, for example, death or sickness of a close family member, financial issues, or job loss, were excluded, due to the fact that such events are considered life stressors causing distress, and thus, they might have led to an overestimation of depressive or anxiety symptoms. Sample size calculation was based on a 6.2% margin of error, 95% confidence level, and 50% estimated prevalence due to absence of studies that reported the prevalence of depressive and anxiety symptoms in PCOS patients; thus, the minimum required sample size was estimated to be 247 subjects.

### 2.3. Data Sources

Data was collected using prevalidated self-administered Arabic version of HAM-A and HAM-D questionnaires [13, 14]. A demographic sheet was given to the participants to evaluate the sociodemographic factors. Hamilton Anxiety Rating Scale (HAM-A) in Arabic version was used to assess the presence of anxiety [13, 15, 16]. It is a fourteen-item questionnaire assessing the intensity of symptoms commonly found in anxiety, such as fears, cardiovascular manifestations relating to anxiousness, sensory somatic complaints, and insomnia. All the items were ranked on five levels Likert scale (0-4). Zero meaning that there were no symptoms experienced, and four reflecting very intense symptoms. The minimum score was zero, and maximum was 56. A score of 14 or more is suggested as the threshold for anxiety symptoms [15]. Hamilton Depression Rating Scale (HAM-D) in Arabic version includes seventeen questions assessing different domains of depression including somatic manifestation, suicidal ideation, agitation, and guilt feelings [14, 15, 17]. First seven items had five levels (0-4), the 8th had four levels (0-3), while the rest had three levels (0-2). The score ranged from 0 to 49. A score of 10 is suggested as the threshold for depressive symptoms [15].

### 2.4. Statistical Analysis

Statistical analysis was performed using Statistical Package for the Social Sciences version 22 (SPSS Inc., Chicago, IL). Reliability testing for both questionnaires was done, HAM-A (14-item) Arabic version had a Cronbach alpha of 0.88, whereas Cronbach alpha for HAM-D (17-item) Arabic version was 0.78, and the Cronbach alpha for the total 31 items was 0.9. Followed by descriptive statics reporting frequencies and percentages for categorical variables such as marital status and monthly income, while mean and standard deviation for continuous variables such as age and duration of infertility. For both HAM-A and HAM-D questionnaires, a total score was computed adding up all the frequencies for the individual items. For HAM-A questionnaire (14 items), the categories were made and scores of <17 for mild anxiety, 18-24 for moderate anxiety, and 25-for severe anxiety. HAM-D questionnaire (17 items) had a score range of 0-49, with a cut-off point of 0-7 for normal symptoms, 8-13 for mild depression, 14-18 for moderate depression, 19-22 for severe, and >23 for very severe depression. Inferential statistics using Chi-square was used to assess the association of sociodemographic characteristics with anxiety and depression. Chi-square test was also used to assess intergroup significance for marital status and anxiety symptoms.

## 3. Results

### 3.1. Demographic Characteristics

A total of 250 patients were recruited for this study with a mean age of 28 ± 8 years and 124 (49%) of them being single. Unemployed participants were 162 (65%), and 27 (11%) have a monthly income lower than 3000 Saudi Riyal and 71 (28%) with a monthly income of 18,000 Saudi Riyal and more. A median of 36 (24-132) months was the duration since PCOS diagnosis for the patients. Among the married participants (*n* = 105), 54 (51%) had conception delay reported post-PCOS diagnosis. (Table 1).

### 3.2. Assessment of Anxiety and Depressive Symptoms

**Figure 1:**
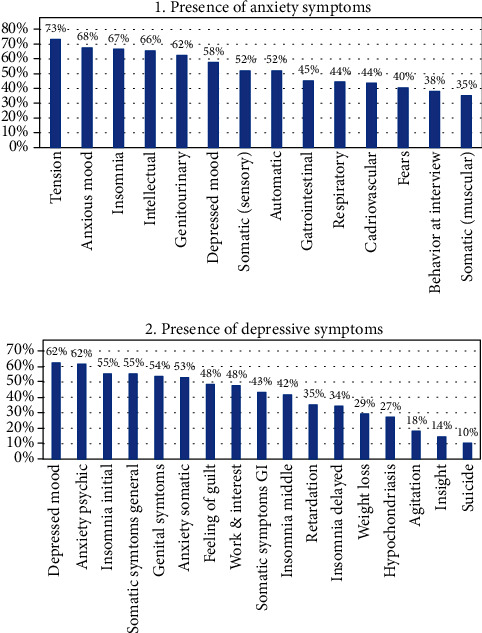
The presence of anxiety and depressive symptoms among women with PCOS.

The depression and anxiety symptoms among PCOS patients were assessed with the Hamilton Depression Rating Scale (HAM-D) and Hamilton Anxiety Rating Scale (HAM-A). A total anxiety score of 13 ± 9 was noted, and out of 250 participants, 184 (77%) had mild anxiety score, and 14 (6%) had severe anxiety. Tension was seen to be the most prominent anxiety symptoms 183 (73%), whereas somatic (muscular) pain was the lowest 88 (35%). Anxiety symptoms were noted in 100 responses (40%) while 150 (60%) had no anxiety symptoms. A total depression score of 10 ± 7 was noted in the study, and within 250 responses, 97 (39%) had a normal score, whereas 11 (4%) had very severe depression. Depressed mood was reported in 154 participants, and psychological anxiety was reported in 156. They were the two most noted depressive symptoms with a percentage of (62%), whereas suicidal ideation was the lowest 26 (10%). Depressive symptoms were reported in 122 participants as opposed to 128 (51%) who had none (Table 2 and Figure 1).

### 3.3. Association of Demographic Characteristics with Anxiety and Depressive Symptoms

Among the demographic variables, marital status, monthly income, and occupational status were statically significantly associated with the presence of anxiety symptoms. Single women were more likely to have anxiety symptoms with 59 (48%) (*χ*2 = 5.8, *p* value <0.01). Furthermore, lower income was associated with a higher prevalence of anxiety symptoms 18 (67%) (*χ*2 = 10.3, *p* value =0.03); additionally, nonemployed women were more likely to have anxiety symptoms compared to employed women 71 (45%) vs. 29 (31%) (*χ*2 = 4.5, *p* value =0.03). For depressive symptoms, none of the sociodemographic indicators showed significant results on cross-tabulation. However, those who experienced conception delay were more depressed than others who did not experience any, showing as a 30 (56%) vs. 19 (37%) with a *p* value of 0.06, demonstrating that the conception delay is borderline significant (Table 3).

### 3.4. Association of Anxiety Symptoms with Marital Status

Out of the 14 anxiety symptoms in HAM-A, six symptoms were found to be significantly associated with single participants. Anxious mood was one of the symptoms found to be significant among single participants 93 (55%) (*χ*2 = 6.151, *p* value =0.013); additionally, somatic muscular symptoms 54 (61%) (*χ*2 = 7.281, *p* value =0.007), gastrointestinal symptoms (*χ*2 = 5.177, *p* value =0.023), fears in 58 (57%) (*χ*2 = 4.152, *p* value =0.042), depressed mood in 83 (58%) (*χ*2 = 8.78, *p* value =0.003), and respiratory symptoms in 63 (57%) (*χ*2 = 4.09, *p* value =0.043) were also reported significantly higher among those women who were single (Table 4).

## 4. Discussion

This study is aimed at determining the prevalence of anxiety and depressive symptoms among PCOS patients and at identifying the possible effect of sociodemographic profile on the prevalence of these symptoms.

More than one-third of the women with PCOS (40%) reported anxiety symptoms, out of which 18% had moderate while 6% had severe. It is theorized that the reason behind this is the nature of their anxiety being secondary to their condition rather than primary. The most common symptom of anxiety was tension, while the least was somatic pain. A possible explanation for the prevalence of anxiety in PCOS women is the physical manifestations of their condition, such as acne, obesity, and hirsutism which lead to negative self-image and low self-esteem [18]. Also, they might experience fear and worries regarding the future and their ability to conceive [18].

In this study, anxiety symptoms were assessed among different socioeconomic factors and were found significantly higher in single women compared to their married counterparts. Studies consistent with this suggested that PCOS symptoms such as obesity, hirsutism, and menstrual irregularities are possibly placing women under the pressure of low self-esteem, negative body image, concerns about future complications, and difficulty finding a life partner, which in turn may increase stress caused by the society regarding marital status and physical appearance [10, 19, 20]. A western study showed that singlehood was a predictor of anxiety due to stressors regarding loneliness, financial support, and social commitments; unfortunately, no local studies were found regarding the singlehood anxiety which might be influenced by our ethnicity [21]. In contrast, a study that assessed quality of life on such women found that there was no impact of menstrual cycle disturbances nor infertility on the mental well-being of the participants; such finding could be attributed to the fact that the mean age of that study is higher than ours; thus, menstruation and fertility concerns are associated mostly with younger age [22].

On further assessment of the patients' anxiety symptoms, single women reported tension and fear as the most prevalent symptoms. Researches suggest that the possible reasons for such findings are the negative reaction from society regarding the patient's physical appearance, android fat distribution, acne, and masculine body hair, which might eventually trigger fear, social phobia, and isolation [19, 23].

Nonemployment and low income were the other socioeconomic factors highly associated with anxiety symptoms among this study's population. Nonemployment was pointed out by literature as a known risk factor for both anxiety and depression [24]. The state of being jobless might affect the individual negatively due to financial and economic difficulties emerging from the lack of income sources. Nonemployment is not only a stressor due to financial obligations but it also gives a sense of low self-esteem and identity issues, since employment helps the person shape their identity, be in continual social interactions, and to earn social ranks that creates the individual's self-image [24].

Depressive symptoms were noted in 122 participants (49%). Similarly, another study that was conducted on women with PCOS to determine the risk of depression found that the prevalence of depression was 40% [25]. The high prevalence of depressive symptoms among PCOS patients could be due to several causes. Such as biological (infertility and premenstrual symptoms) or hormonal factors. Contrarily, a study which was conducted on 120 PCOS patients reported that 68.3% were nondepressed [26]. The variation in results could be due to the differences in cultural and ethnic backgrounds. Seeing that in Middle Eastern patriarchal society women are placed under a higher social pressure regarding marriage and childbearing. In this current study, mild to moderate depression scores were the most prevalent with a percentage of 30% for mild and 19% for moderate. On the other hand, a meta-analysis found that 11 studies reported a high prevalence of moderate to severe depressive symptomatology among PCOS women [27].

Fatigue and sleep disturbances were the most reported depressive symptoms in a study conducted on 103 women with PCOS, while in this current study, it was found that depressed mood and psychological anxiety were the two most prominent symptoms with a percentage of 62% [28]. Suicidal ideation was found to be the least reported depressive symptom with a percentage of 10%. On the other hand, a case-control study conducted in Sweden to assess the relationship between PCOS with psychiatric symptoms and disorders found that suicidal attempts were significantly seven times higher compared to the controls [29]. The difference between the suicidality rate in Sweden and this current study might be due to the influence of Islam on Muslim's values and beliefs regarding the forbiddance of committing suicide [30].

On cross tabulation, none of the sociodemographic indicators showed significant results for depressive symptoms. However, PCOS participants who experienced conception delay had more depressive symptoms than others who did not experience any, showing as 30 (56%) vs. 19 (37%), respectively. This result is also shown in a study that was conducted in Italy comparing two groups of infertile subjects with two fertile groups using the Hamilton Depression Rating Scale [31]. Both of the infertile group scores were significantly higher than the control groups with an average of above the cutoff scores for mild depression [31]. Infertility caused by PCOS could trigger stress and psychological issues such as distress, social maladjustment, and loss of control [32]. For some women, having children is an important part of their female identity; thus, infertility affects their quality of life as well as their partner and family. Moreover, infertility is often associated with lower self-esteem, low social status, divorce, and job dissatisfaction [33]. It is noted that the effect of infertility on women depends on socio-cultural factors, traditions, and religious beliefs [34]. A study was done on Austrian women and Muslim immigrants in Vienna to assess the influence of socio-cultural (religious) background on health-related quality of life on PCOS women with infertility [34]. Austrian women reported that infertility is not as much of an obstacle as it is for Muslim women [34].

There were a number of limitations present in the study, one of which was the study type being cross-sectional. It limited the full evaluation of all PCOS aspects and its future drawbacks regarding patient's psychology. Moreover, a methodology limitation was the use of questionnaires, resulting in response bias, selection bias, and the application of only screening tools that limited the full evaluation anxiety and depression, so the results were interpreted in the context of anxiety and depressive symptoms only, which was also due to the absence of a psychologist consultation. Moreover, some literature suggested that patients' BMI and hormonal profile are a possible cause of their mental symptoms; thus, the lack of these measurements in this study was considered as a limitation. Also, the study being conducted in one center and the lack of a control group were considered limitations due to the inability to acquire a comprehensive picture regarding anxiety and mood disorders in PCOS population in Riyadh.

## 5. Conclusion

The overall prevalence of depressive symptoms in women with PCOS was 49%, compared to anxiety symptoms which were present among 40%. Single marital status and unemployment were predictors of anxiety among women with PCOS. Clinicians should be aware of the high risk of developing anxiety or depression syndromes among these women and screen them regularly for the presence of said symptoms. Appropriate pharmacological treatment alongside proper psychotherapeutics and psychosocial support should be included in the treatment plan.

## Figures and Tables

**Table 1:** Sociodemographic and disease profile of the participants (*n* = 250).

Variables	Categories	Count	%
Age	Mean ± SD	28 ± 8	

Nationality	Saudi	240	96%
	Non-Saudi	10	4%

Marital status	Single	124	49%
	Married	117	47%
	Other	9	4%

Children (*n* = 126)	No	44	18%
	Yes	81	32%

Educational level	No education	1	0%
	Elementary	1	0%
	Middle	2	1%
	High school	51	21%
	Bachelor	181	73%
	Higher degree	11	5%

Occupational status	Nonemployed	162	65%
	Employed	88	35%

Monthly income	<3000 SR	27	11%
	3000 to 7000 SR	46	18%
	8000 to 12000 SR	71	28%
	13000 to 17000 SR	35	14%
	18000 SR and more	71	28%

Duration since diagnosis in months	Median (Q1-Q3)	36 (24-132)	

Px taking PCOS treatment	Yes	131	58%
	No	94	42%

PCOS treatment duration in months	Median (Q1-Q3)	12 (6-96)	

Presence of conception delay (*n* = 105)		54	51%

Duration of conception delay in years	Mean ± SD	5 ± 3	

Pregnancy after delayed conception (*n* = 56)		24	43%

The use of assisting techniques for conception (*n* = 22)		18	82%

**Table 2:** Prevalence of anxiety and depressive symptoms among women with PCOS (*n* = 250).

Variables	Categories	*N* (%)
Total score anxiety	Mean ± SD Median (Q1-Q3)	13 ± 9 10 (6-29)

Total score depression	Mean ± SD Median (Q1-Q3)	10 ± 7 9 (6-22)

Anxiety (score ≥ 14)	Absent	150 (60%)
	Present	100 (40%)

Anxiety categories	Mild anxiety (score ≤ 17)	184 (77%)
	Moderate anxiety (score 18-24)	42 (18%)
	Severe anxiety (score 25-30)	14 (6%)

Depression (score ≥ 10)	Absent	128 (51%)
	Present	122 (49%)

Depression categories	Normal (score 0-7)	97 (39%)
	Mild depression (score 8-13)	75 (30%)
	Moderate depression (score 14-18)	48 (19%)
	Severe depression (score 19-22)	19 (8%)
	Very severe depression (score ≥ 23)	11 (4%)

**Table 3:** Association of anxiety and depression among patients with PCOS with sociodemographic profile (*n* = 250).

		Anxiety				*p* value	Depression				*p* value
		Absent		Present			Absent		Present		
		*N*	%	*N*	%		*N*	%	*N*	%	
Marital status	Single	65	52%	59	48%	0.01^∗^	57	46%	67	54%	0.10
	Married	85	68%	41	33%		71	56%	55	44%	

Conception delay	No	35	69%	16	31%	0.17	32	63%	19	37%	0.06
	Yes	30	56%	24	44%		24	44%	30	56%	

Children	No	27	61%	17	39%	0.29	24	55%	20	46%	0.77
	Yes	58	71%	24	29%		47	57%	35	43%	

Monthly income	<3000 SR	9	33%	18	67%	0.03^∗^	11	41%	16	59%	0.66
	3000 to 7000 SR	26	57%	20	44%		21	46%	25	54%	
	8000 to12000 SR	46	65%	25	35%		38	54%	33	47%	
	13000 to 17000 SR	24	69%	11	31%		19	54%	16	46%	
	18000 SR and more	45	63%	26	37%		39	55%	32	45%	

Occupational status	Non-employed	86	55%	71	45%	0.03^∗^	74	47%	83	53%	0.08
	Employed	63	69%	29	32%		54	59%	38	41%	

PCOS drug	No	78	60%	53	41%	0.53	69	53%	62	47%	0.31
	Yes	52	55%	42	45%		43	46%	51	54%	

Educational status	High school or less	29	53%	26	47%	0.22	29	53%	26	47%	0.77
	Bachelor or higher	119	62%	73	38%		97	51%	95	50%	

^∗^The Chi-square statistic is significant at the 0.05 level.

**Table 4:** Association of anxiety symptoms with marital status (*n* = 250).

Anxiety symptoms	Single (*n* = 124)		Married (*n* = 126)		*p* value
	*N*	%	*N*	%	
Anxious mood	93	55%	76	45%	0.013^∗^
Tension	99	54%	84	46%	0.019^∗^
Fears	58	57%	43	43%	0.042^∗^
Insomnia	83	50%	83	50%	0.788
Intellectual	77	47%	86	53%	0.266
Depressed mood	83	58%	61	42%	0.003^∗^
Somatic (muscular)	54	61%	34	39%	0.007^∗^
Somatic (sensory)	63	49%	67	52%	0.708
Cardiovascular symptoms	54	50%	55	51%	0.987
Respiratory symptoms	63	57%	48	43%	0.043^∗^
Gastrointestinal symptoms	65	58%	48	43%	0.023^∗^
Genitourinary symptoms	82	53%	74	47%	0.227
Autonomic symptoms	61	47%	69	53%	0.378
Behavior at interview	56	59%	39	41%	0.021^∗^

^∗^The Chi-square statistic is significant at the 0.05 level.

## Data Availability

Data is available from the principal investigator upon request. However, it cannot be shared online as per data sharing restrictions from the approving body.
